# Enhanced photothermoelectric conversion in self-rolled tellurium photodetector with geometry-induced energy localization

**DOI:** 10.1038/s41377-024-01496-0

**Published:** 2024-07-04

**Authors:** Jiayuan Huang, Chunyu You, Binmin Wu, Yunqi Wang, Ziyu Zhang, Xinyu Zhang, Chang Liu, Ningge Huang, Zhi Zheng, Tingqi Wu, Suwit Kiravittaya, Yongfeng Mei, Gaoshan Huang

**Affiliations:** 1 Department of Materials Science & State Key Laboratory of Molecular Engineering of Polymers, Fudan University, Shanghai, 200438 China; 2 Yiwu Research Institute of Fudan University, Yiwu, 322000 Zhejiang China; 3 International Institute of Intelligent Nanorobots and Nanosystems, Fudan University, Shanghai, 200438 China; 4 ShanghaiTech Quantum Device Lab, ShanghaiTech University, Shanghai, 200120 China; 5 Department of Electrical Engineering, Faculty of Engineering, Chulalongkorn University, Bangkok, Thailand; 6 Shanghai Frontiers Science Research Base of Intelligent Optoelectronics and Perception, Institute of Optoelectronics, Fudan University, Shanghai, 200438 China

**Keywords:** Optoelectronic devices and components, Optical materials and structures, Integrated optics

## Abstract

Photodetection has attracted significant attention for information transmission. While the implementation relies primarily on the photonic detectors, they are predominantly constrained by the intrinsic bandgap of active materials. On the other hand, photothermoelectric (PTE) detectors have garnered substantial research interest for their promising capabilities in broadband detection, owing to the self-driven photovoltages induced by the temperature differences. To get higher performances, it is crucial to localize light and heat energies for efficient conversion. However, there is limited research on the energy conversion in PTE detectors at micro/nano scale. In this study, we have achieved a two-order-of-magnitude enhancement in photovoltage responsivity in the self-rolled tubular tellurium (Te) photodetector with PTE effect. Under illumination, the tubular device demonstrates a maximum photovoltage responsivity of 252.13 V W^−1^ and a large detectivity of 1.48 × 10^11^ *Jones*. We disclose the mechanism of the PTE conversion in the tubular structure with the assistance of theoretical simulation. In addition, the device exhibits excellent performances in wide-angle and polarization-dependent detection. This work presents an approach to remarkably improve the performance of photodetector by concentrating light and corresponding heat generated, and the proposed self-rolled devices thus hold remarkable promises for next-generation on-chip photodetection.

## Graphical abstract

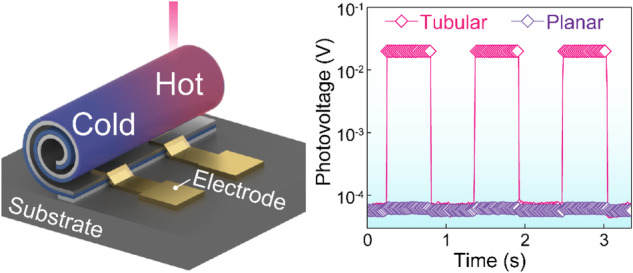

## Introduction

Light is an important information transport intermediary. Broader conceptualizations of communication signal receivers can be considered as a type of photodetector^1^. Generally, the photodetector is defined as a signal transformation device that can receive a photonic signal and convert it into an electronic signal. To meet the demand for miniaturization, on-chip photodetectors are brought to the spotlight due to their wide applications in optical communication, computing circuits, imaging, and materials analysis^2–5^. Traditional photodetectors possess superior properties of photoresponse^6,7^, but their response spectra are primarily constrained by the bandgaps of the materials used^8–10^. Hence, thermal detectors are supposed to be potent devices for broadband detection, driven by the temperature-sensitive characteristic of active materials^11^.

The study of the photothermoelectric (PTE) effect, induced by temperature diversity, has garnered recent attention, mostly in low-dimensional materials, including nanorods, nanotubes, and two-dimensional (2D) materials^12–15^. The PTE effect consists of two processes: photothermal conversion and thermoelectric effect^16^. During the photothermal conversion, photons are absorbed by the materials, leading to the generation of a temperature difference (*∆T*)^16^. For the thermoelectric effect, namely the Seebeck effect, an electric potential is established as carriers diffuse from the hot end to the cold end. Assuming the device maintains a constant Seebeck coefficient (*S*) under one-dimensional temperature variation, the photovoltage (*V*_ph_) of detector can be described as *V*_ph_ = *S*Δ*T*^17^. Considering the operational mechanism of the PTE effect, factors such as photon collection efficiency, Seebeck coefficient, and constructed temperature difference play important roles.

Generally, the key factors to obtaining a higher responsivity in PTE detectors are promoting photon absorption, widening the diversity of the Seebeck coefficient, or increasing temperature difference. To achieve effective localized light absorption, optical absorbers such as antennas, plasmonic nanostructure, and metamaterials, are utilized^18–20^. Whereas, the introduction of additional sophisticated structures increases fabrication complexity. To diversify the Seebeck coefficient, producing p-n junctions through doping or using different ohmic contacts has been applied, which can risk the consistency of the active materials^19,21,22^. Moreover, to increase the temperature difference, the focused light beam is a common method to generate localized absorption, which must be constricted by the illumination system^23^. In addition, all the above methods have been studied based on the planar lateral or vertical device. Energy localization in 3D micro/nano structures may effectively enhance the PTE energy conversion, but related studies are still scarce. Understanding the interaction mechanism of multiple energy fields in 3D structures is vital for high-performance PTE detectors and thus needs further investigation.

For the geometry of the device, 3D micro/nano structures provide ideal platforms to separate the device from the substrate, avoiding the influence of irrelevant components^24^. Compatible with practical chip manufacturing, self-rolling of the nanomembrane can construct 3D tubular structure with various materials for advantageous applications^25^. Specifically, previous investigations demonstrate that self-rolled structures can be used as optical microcavities to confine electromagnetic field energy^26^. The high-quality resonance and the internal reflection therein may lead to enhanced light absorption^27^. From the thermal perspective, the self-rolled structures can localize the thermal energy in the 3D tubular geometries and inhibit heat diffusion to the substrate^28^. Thus we consider the self-rolled structures to be conducive to PTE detectors as they can serve as good light absorbers and heat retainers with tunable geometry.

In the present work, we construct a self-rolled tubular Te-based detector (TTD) to enhance the PTE effect and corresponding photodetection performance. On the basis of theoretical simulation, we study the multiple-field interaction by confirming the light trap effect and thermal insulation within the tubular structure. To firmly verify the PTE effect in the TTD, the relationship between electrical/optoelectronic property and irradiation position is meticulously explored. Moreover, we investigate the influence of geometric factors by fabricating self-rolled structures with different numbers of rotations to tune the light trap effect. Our TTD demonstrates a good photoresponse from visible to infrared region with a maximum responsivity of 252.13 V W^−1^ and maximum detectivity of 1.48 × 10^11^ *Jones*, substantially outperforming those of planar device (i.e., PTD, planar Te-based detector). Furthermore, our TTD with a tubular geometry possesses an ultrawide-angle detection capability and presents a stable broadband polarization-dependent photoresponse. In this work, we disclose the mechanism of localization and conversion of multiple energies in the 3D tubular device and propose an approach to design and fabricate high-performance PTE detectors by a nanomembrane self-rolling process. The output thus broadens the fresh horizon in next-generation on-chip optoelectronic devices.

## Results

### Design concept and realization of TTD

**Fig. 1:**
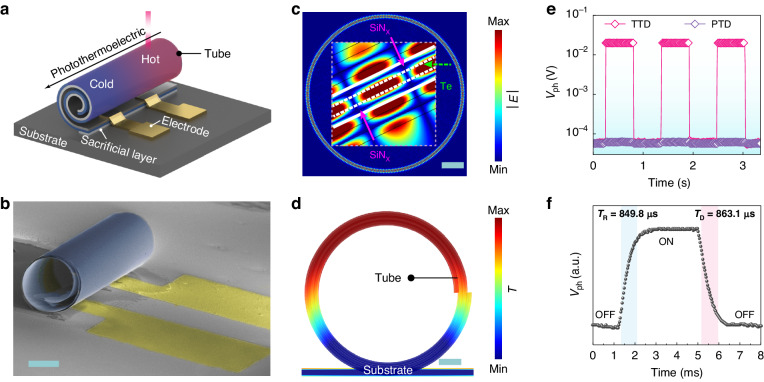
Structure design and implementation of TTD. **a** Schematic diagram of the operation approach and components in TTD. **b** SEM image of a TTD device. Scale bar, 20 μm. **c** Simulated electric field distribution. Inset: the enlarged view of the field distribution in the multi-layered wall. Scale bar, 5 μm. **d** Simulated thermal distribution in the tube and substrate. Scale bar, 5 μm. **e** *V*_ph_-*t* curves of the TTD and the PTD with pulsed illumination of 940 nm laser at zero bias. **f** Photoresponse time of the TTD irradiated by 940 nm laser

In this work, we constructed a 3D tubular structure as a PTE detector via a nanomembrane self-rolling process. The tubular structure is an excellent optical absorber due to the light trap effect inside the tube wall^27^. From the cross-sectional view, a tube can also be considered as a ring with a dot linking to the substrate, allowing for minimizing heat loss from the structure to the substrate. Therefore, the self-rolled tubular structure can magnify the temperature difference owing to the enhanced light absorption and thermal localization. In addition, we selected Te layer prepared by magnetron sputtering as the active material due to its high *S* of 2672.72 μV K^−1^ at room temperature (Table S1) and high Hall mobility of 588 cm^2^ V^−1^ s^−1^. Besides, its feasible deposition approach is compatible with self-rolling and chip manufacturing processes. Figure 1a schematically illustrates the structure of the TTD and its operation approach. The core component, TTD, includes a multi-layered self-rolled tubular structure (the self-rolled nanomembrane consists of a Te layer, a pre-strained layer, and a protection layer), electrodes (part of which are rolled up into the tube), a sacrificial layer, and a substrate. Specifically, we deposited alumina (Al_2_O_3_) as a protection layer, palladium (Pd)/gold (Au) as electrodes, silicon nitride (SiN_x_) as a pre-strained layer, germanium (Ge) as a sacrificial layer, and thermally oxidized silicon wafer as a substrate. Subsequently, the TTD was produced by selectively etching away the sacrificial layer and self-rolling driven by the stress gradient inside the SiN_x_ layer (flow chart in Figure S1)^25^. Scanning electron microscopy (SEM) images in Figs. 1b and S2 affirm that the tubular device comprises the self-rolled microtube and electrodes. In the current approach, the high-stress difference in SiN_x_ layer (Figure S3) ensures a reliable rolling process, and the image in Figure S4 demonstrates the high yield of uniform TTDs, which helps to achieve good reproducibility of detection performance.

Our experimental result indicates that both photothermal conversion and thermoelectric effect occur upon light irradiation of the TTD and the PTE conversion is greatly enhanced due to the energy localization in this unique geometry. To further investigate optical absorption in the TTD, a simplified model of the tube with sandwich “SiN_x_-Te-SiN_x_” layers in the wall is built up since the protection layer is thin enough (typically ~ 5 nm) to be neglected. The corresponding simulated field distribution shown in Fig. 1c depicts evident whispering gallery mode (WGM) in the tube wall at 940 nm, proving light energy localization therein^26^. During resonance, light can be more effectively trapped in the tube wall, with most of the light energy concentrating in the Te layer (green arrow) rather than in the SiN_x_ layer (pink arrow), as illustrated in the inset of Fig. 1c. One probable explanation is that when photons get into the multi-layered tube wall, they prefer to stay in the Te layers with higher refractive index rather than pass into the SiN_x_ layers with lower refractive index. Experimentally, resonance mode with a high-quality factor (Q factor) is supported in the TTD, as evident in the photoluminescence (PL) spectra (Figure S5). The resonance peak at ~940 nm demonstrates a Q factor up to 624, and the Q factor of the peak at ~1100 nm is 620, suggesting a strong light trap effect in the tube wall. The simulated field distributions of WGM at different wavelengths in Figure S6 reveal that the optical localization is enhanced across a wide wavelength range. In addition, as shown in Figure S7, the existence of an air gap between adjacent rotations (which is closer to the real situation) should weaken the light trap effect due to the scattering at the interface, but the preference for light energy localization in the Te layers with higher refractive index can still be observed.

Besides, we simulated the temperature distributions both in the cross-section of the tube and along the tube axis to explore the thermal energy localization of the TTD. For the cross-section, the simulated result clearly reveals significant heat localization in the tube wall (Fig. 1d), indicating that the special configuration of TTD leads to thermal energy localization. Figure S8 specifically demonstrates the temperature distribution along the tube axis. The thermal energy distribution and the produced temperature difference from the illuminated spot to the far end can be easily distinguished, which is conducive to the PTE effect. When it comes to practical application with an illumination of 940 nm laser at a selected position (corresponding to spot 2 in Figure S8), the photovoltage under zero bias in Fig. 1e exhibits a notable boosting in TTD, which is ~ 307 times higher than that in PTD. Therefore, the TTD is considered an ideal platform to enhance the PTE effect for further application. Moreover, our TTD breaks the response speed limit in thermal photodetectors, which is typically in the order of milliseconds^29^. For our TTD, rise time (*τ*_R_) and decay time (*τ*_D_) are determined to be 849.8 and 863.1 μs respectively, as shown in Fig. 1f. The bandwidth-response curve in Figure S9 also confirms the validity of measured response time (Note S1). The fast response speed also serves as evidence of the hot-carrier-assisted mechanism in our TTD^16^, as heat is efficiently carried and transferred by hot carriers, which bypasses the lattice overheating associated with phonon-transported mechanisms in traditional devices^30^.

### Microstructural characterizations of TTDs

**Fig. 2:**
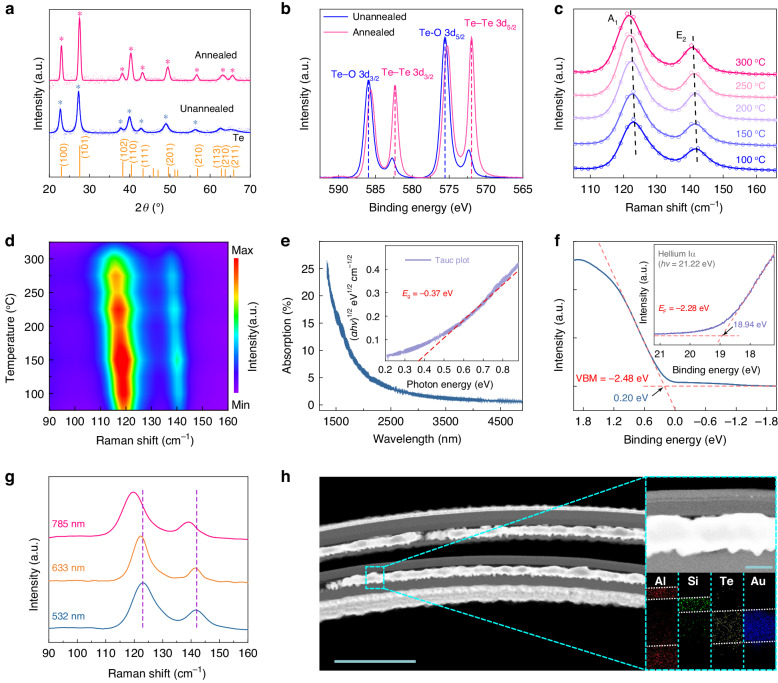
Microstructural characterizations of TTD. **a** XRD patterns of Te layers before (blue line) and after (pink line) being annealed at 150 °C. Te standard PDF card No. 00-036-1452 (orange line) is also shown for comparison. **b** XPS spectra of Te layers before (blue line) and after (pink line) being annealed at 150 °C. **c** Raman spectra of Te layers annealed at different temperatures ranging from 100 to 300 °C. **d** In situ Raman spectra of Te layer with high-temperature treatment. **e** FTIR spectrum of Te layer. Inset: Tauc plot indicates the indirect interband transition for Te layer. **f** UPS spectrum of the valence-band region of Te layer. Inset: secondary electron cutoff spectrum of Te layer. **g** Raman spectra of Te layer under the excitations of 785, 633, and 532 nm. **h** Cross-sectional HAADF image of a cut of TTD. Scale bar, 1 μm. Upper inset: enlarged HAADF image of the TTD. Scale bar, 100 nm. Lower inset: corresponding elemental mapping result

TTD is an integrated device consisting of different materials, with the Te layer playing a key role in absorbing optical energy and transferring it to an electrical signal through a photothermoelectric conversion. Thus, the microstructural properties of the Te layer are carefully characterized. Here, the Te layer was deposited by magnetron sputtering, and annealed by rapid thermal annealing. The corresponding atomic force microscopy (AFM) characterization in Figure S10 evinces the impressive uniformity of the Te layer before annealing. The crystalline structure of Te layer was further probed by X-ray diffraction (XRD) patterns (Fig. 2a), wherein the impact of post-treatment was also explored. Compared to the unannealed sample, the sharper peaks demonstrate that the Te layer annealed at 150 °C owns larger crystal grains. Additionally, X-ray photoelectron spectroscopy (XPS) results (Fig. 2b) indicate the predominant evolution from the Te-O state to the Te-Te state during annealing^31^. Besides, the testing results show typical first-order Raman active mode of Te layer, including characteristic peaks at 123 cm^−1^ for A_1_ mode and 141 cm^−1^ for E_2_ mode and Figure S11 reveals that the peaks blueshift after annealing. More precisely, we figure out that the peaks shift to low wavenumber with the escalating annealing temperature from 100 to 300 °C (Fig. 2c), possibly induced by the non-crystal phase change^32^. To further investigate the evolution of Te layer with increased annealing temperature, in situ Raman spectroscopy was conducted, and the results are shown in Fig. 2d. The experimental results demonstrate that the Raman peaks of Te layer are noticeable at temperatures up to ~ 300 °C, indicating a high thermal stability^32^. Therefore, we determined the annealing temperature of 150 °C as the optimized parameter, which can maintain the balance between the increased grain size and limited non-crystal phase change. To deeply explore the band structure of Te layer, the absorption spectrum was measured by Fourier transform infrared spectrometer (FTIR). Figure 2e shows that the absorption edge of Te layer is around 3350 nm, while the corresponding Tauc plot in the inset indicates the optical bandgap is ~ 0.37 eV (Note S2). The electronic structure of Te layer was then confirmed by ultraviolet photoelectron spectroscopy (UPS). The secondary electron cutoff spectrum in the inset of Fig. 2f displays that the Fermi level (*E*_F_) is -2.28 eV. Based on the valence band spectrum in Fig. 2f, the valence band maximum (VBM) and conduction band minimum (CBM) of Te is determined to be -2.48 and -2.11 eV, respectively (Note S3), and the band structure of Te layer is schematically illustrated in Figure S12.

We notice that the self-rolled structure may possess significant polarization dependence^33^, and thus the current samples are investigated in detail by using polarized Raman spectroscopy. For flat Te layer, it is worth noting that only A_1_ mode (121 cm^−1^, corresponding to in-plane chain expansion) expresses obvious intensity alteration with polarization angle (Figure S13), consistent with the results in previous literature on bulk Te^34^, while no obvious intensity evolution can be observed in other Raman modes. However, for the TTDs, polarization-dependent features of the active Raman modes, including E_1_ mode at 98 cm^−1^ and E_2_ mode at 139 cm^−1^ were identified, as represented in Figure S14. This is probably caused by the strain generated in Te layer during the rolling process. Driven by the underneath pre-strained SiN_x_ layers, compressive strain is introduced into Te layer on the top of SiN_x_ layer during rolling, while the whole multi-layered nanomembrane rolls up to form a tubular structure^35^. The strain in the Te layer should not be uniform along the depth and it can be evaluated by Raman spectra with different excitation wavelengths^36^. As the excitation wavelength increases, the Raman characteristic peaks shift to lower wavenumbers (Fig. 2g), indicating the lattice expansion^37^. The compressive strain of Te layer induced by the rolling process is roughly estimated to be ~ 0.35-0.50% according to the Raman spectrum measured with a 532 nm laser (Note S4). This compressive strain is expected to increase with depth, as demonstrated by the larger shift in the spectrum obtained with a 785 nm laser.

To clearly confirm the microstructure of TTDs, we deposited a 50 nm Al_2_O_3_ layer by atomic layer deposition (ALD) to strengthen the structure and prepared a top cut of the TTD by using focused ion beam (FIB). Scanning transmission electron microscopy (STEM) was applied to check the multi-layered structure of the TTD. The high angle annular dark field (HAADF) image of the cross section shown in Fig. 2h displays a curved multi-layered structure. Accordingly, the sophisticated elemental mapping by energy-dispersive X-ray spectroscopy (EDS) of the enlarged portion in the upper inset is displayed in the lower inset. Every rotation of the tube wall contains Al_2_O_3_ as the protection layer, SiN_x_ as the pre-strained layer, Te as PTE material, and Pd/Au as the electrodes. The mixture coloration of Te and Au should be probably caused by the melting of Te under the high temperature during the FIB cutting process^38^.

### Verification of the PTE effect of the TTD

**Fig. 3:**
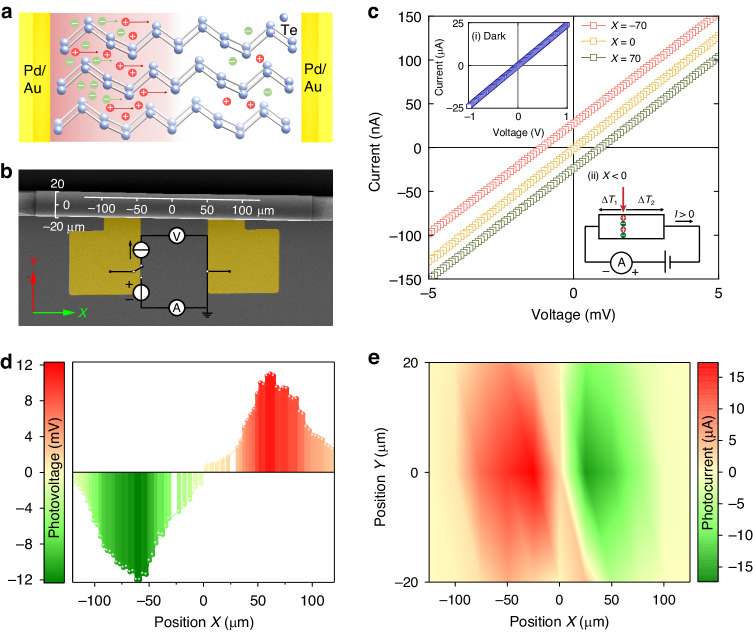
Verification of the PTE effect in the TTD. **a** Schematic diagram shows that the carriers (green and red dots represent electrons and holes, respectively) diffuse from the hot region to the far end. **b** SEM image of a TTD device with the coordinates system. **c** Measured *I-V* curves of the TTD under 940 nm laser illumination at three different positions: *X* = -70 μm (red square), *X* = 0 (yellow square), and *X* = 70 μm (green square) at the same light power density of 1500 μW cm^−2^. Inset panels: (i) Measured *I-V* curve of TTD without laser illumination; (ii) Schematic diagram of current flow in TTD under zero bias when the laser illumination spot at *X* < 0. **d** Line scan of photovoltage along the axial axis (*X* direction). **e** Corresponding photocurrent mapping results

In Fig. 3a, we schematically describe the energy conversion process within the device due to the PTE effect with the assistance of hot carriers. Once the laser irradiates the device, an inevitable temperature difference arises from the illuminated spot to the far end, and carriers (electron-hole pairs) are generated by the inspiration of local temperature difference. Here, Te is a p-type semiconductor (*S* > 0 in Table S1), so holes (red dots) diffusion from the hot area (left) to the cold area (right) is dominant^39,40^. Thus, a nonequilibrium dynamic effect is developed due to the heat generated in PTE materials by the incoming photons from the laser, which then leads to an electrical potential variation. When the light turns off, the equilibrium is gradually restored, and the photoresponse fades away^16,39^.

To further investigate the PTE effect in the TTD, we carried out a set of experiments as follows. For the sake of clear description, the coordinate system is marked in the SEM image (Fig. 3b) and thus the position on the TTD can be easily labelled. ∆*T* is defined as the temperature on the right side subtracting that on the left side. As described in the inset panel (i) of Figs. 3c and S15, the linear dark *I-V* curve with the applied voltage increasing from -1 to 1 V demonstrates the ohmic contact of the device, which is different from the characteristic curve of Schottky contact^40^. Moreover, since the photoresponse is irrelevant to the bandgap of the PTE material, illumination does not change the slope of the *I-V* curve; instead, it only shifts the curve along the vertical direction^16^. During the experiment, light continuously illuminates the TTD at a specific spot, generating an additional voltage by temperature difference and corresponding PTE effect. For instance, as illustrated in the inset panel (ii) of Fig. 3c, when a spot at *X* < 0 is illuminated, the temperature difference on the left side (|∆*T*_1_|) is smaller than that on the right side (|∆*T*_2_|), producing different electrical potentials on the two sides. When the applied bias voltage is 0, this electrical potential difference acts as an extra source, providing current flow along the positive direction (*I* > 0). The *I-V* curve consequently shifts upwards, deviating from the zero point. When illumination moves to a spot at *X* > 0, the curve shifts oppositely. When the light spot is located at *X* = 0, temperature variations are equal on both sides (|∆*T*_1_| = |∆*T*_2_|), and no potential difference can be obtained. Under such circumstance, the *I-V* curve passes the origin of the coordinate. Experimentally, Figs. 3c and S16 clearly manifest this phenomenon.

Furthermore, these experimental results suggest that the current TTD is a self-driven detector, and self-driven voltage *V*_ph_ is measured without any external power supply and the illumination position is altered. Figure 3d shows the evolution of *V*_ph_ when the illumination spot is moved along the axial direction of the tubular structure. A significant elevation of |*V*_ph_| is observed in the position range of *X* = (-79, -49) and *X* = (49, 77), where the |*V*_ph_| exceeds 80% of the peak value. This is because that the semiconductor-metal interface provides extra heat due to the light absorption in electrodes^41^. Here, the self-driven performance of PTD was also characterized for comparison. Along the horizontal direction, the PTD demonstrates a similar evolution, confirming a similar PTE effect in the Te layer (Figure S17). Figure 3e further exhibits a photocurrent mapping for TTD with light spots moving along both *X* and *Y* directions to demonstrate the influence of the tubular geometry. Similar to our previous discussion, photocurrent is negative at *X* > 0, and positive at *X* < 0. Moreover, the best optoelectronic response arises at *Y* = 0, meaning the best response performs when light incidents along the diameter from the top. In addition, we varied the wavelength of light to study its impact on the *I-V* curve (Figure S18). Apart from the same shift discussed above, it is worth mentioning that the shift changes at different illumination wavelengths. The shift is the largest when the device is irradiated by a 940 nm laser, again signifying the prominent localization of optical and thermal energies (Figs. 1d and S5).

### Self-driven photodetection performances of TTD

**Fig. 4:**
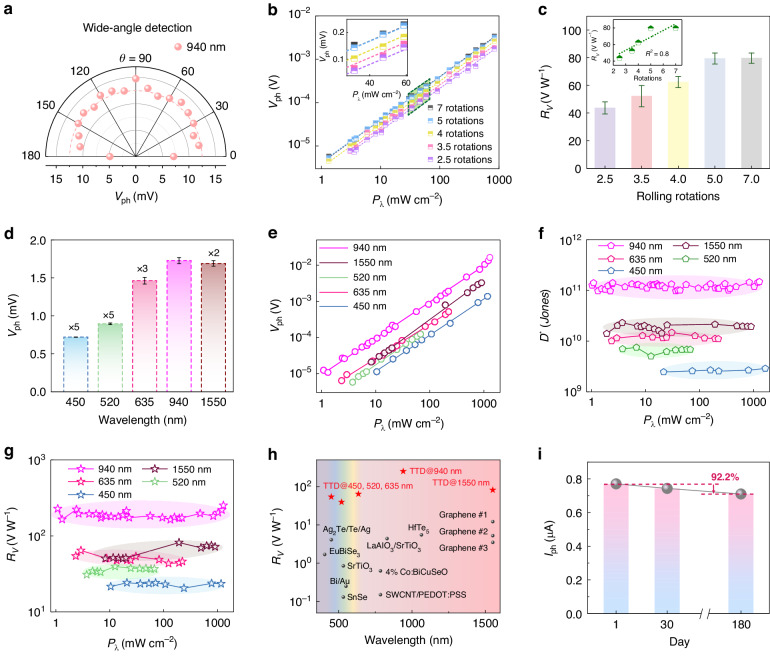
Self-driven photodetection performances of TTD. **a** Measured *V*_ph_ under light illumination at different incident angles. **b** *V*_ph_ as a function of *P*_*λ*_ for TTD with different rolling rotations. Inset: enlarged panel of the green region. **c** Statistics of the corresponding *R*_*V*_ of TTD with different rolling rotations. Inset: linearly fitted correlation curve between rolling rotations and *R*_*V*_. **d** Measured *V*_ph_ of the TTD under different wavelengths of 450, 520, 635, 940, and 1550 nm. **e–g** *V*_ph_, D^*^, and *R*_*V*_ as functions of *P*_*λ*_ for TTD illuminated by lasers of 450, 520, 635, 940, and 1550 nm. **h** Comparison of the *R*_*V*_ of our TTD and other self-driven photodetectors based on the PTE effect. The references are listed in Table S2. **i** *I*_ph_ of the TTD illuminated by 940 nm after 1, 30, and 180 days of exposure in the air atmosphere at room temperature

The TTD possesses a uniform circular cross section which is supposed to support wide-angle detection^42^. Therefore, employing a homemade rotation system where the incident laser can rotate around the device, we characterized the angle-resolved photoresponse and investigated the orientation dependence of TTD, as displayed in Figure S19. Set the angle between the laser and substrate as θ, the angle-dependent photoresponse with 940 nm illumination is described in Fig. 4a. The result attests that for incident light at different angles, TTD exhibits an excellent omnidirectional characteristic of photodetection and the obtained *V*_ph_ is independent of incident angle ranging from ~ 10° to ~ 170°. In the present work, responsivity (*R*) and detectivity (D^*^) are introduced to evaluate self-driven photodetection performance quantitatively. Here, *R* for voltage (*R*_*V*_), *R* for current (*R*_*I*_), and D^*^ are respectively defined by^43,44^:

$${R}_{V}=\frac{{V}_{{\rm{ph}}}}{{P}_{\lambda }A}$$

$${R}_{I}=\frac{{I}_{{\rm{ph}}}}{{P}_{\lambda }A}$$

and

$${{D}^{*}}=\frac{{R}_{I}\sqrt{A}}{\sqrt{2e/{I}_{{\rm{dark}}}}}$$

where *I*_ph_ is photocurrent, *P*_*λ*_ is the incident power density, *A* is the effective illumination area, *e* is the electron charge, and *I*_dark_ is the current at dark state. To explore the function of the multi-rotation wall of the TTD, we fabricated TTDs of different rolling rotations with similar diameters of ~ 32 μm (the influence of the rolling rotations on the diameter can be neglected because of the small thickness of the nanomembrane), and compared their photodetection performances. With the increased number of rotations, TTD yields higher *V*_ph_ and D^*^ under the same laser power density, as depicted in Figs. 4b and S20. Under the illumination of a 940 nm laser, *R*_*V*_ represents a near-linear relationship with the number of rotations (Fig. 4c), and the estimated fitting coefficient of determination (*R*^2^ = 0.8) is close to 1 (inset of Fig. 4c). This can be explained by the light confinement in the tube wall with the sandwich structure^45^, as the optical energy localization in the wall mostly relies on the number of rotations. Simulated results in Figure S21 confirm this by demonstrating a linear relationship between the electrical field intensity in tube walls with different rolling rotations. As rolling rotations increase, more photons are collected in the tube wall, amplifying that the generation of heat and the localization of energies contribute to increased *V*_ph_ and augmentation of *R*_*V*_.

As a PTE detector, the TTD is supposed to be a favorable broadband detector. To prove this, *V*_ph_ under light with different wavelengths ranging from 450 nm to 10.6 μm were measured and the typical results are depicted in Figs. 4d and S22, preliminarily demonstrating the broadband sensing ability. Since the incident power intensities of lasers with different wavelengths are not the same, further characterization is required. Relationships between *V*_ph_/*I*_ph_ and *P*_*λ*_ under different illumination wavelengths indicate good linearity (Figs. 4e and S23a). At 940 nm in the infrared region, the TTD presents a remarkable performance, e.g., *R*_*I*_ of 4.69 mA W^−1^ (Figure S23b), D^*^ of 1.48 × 10^11^ *Jones* (Fig. 4f), and *R*_*V*_ of 252.13 V W^−1^ (Fig. 4g). It is worth noting that these key indicators of photodetection exhibit quite good consistency at both weak and strong light illuminations. Specifically, we get the highest *R*_*V*_ of 81.62 V W^−1^ at 1550 nm, and *R*_*V*_ of 64.24 V W^−1^ at 635 nm. Under the irradiation of light with shorter wavelengths, the *R*_*V*_ is 39.52 V W^−1^ at 520 nm, and 24.17 V W^−1^ at 450 nm. Here, the variation in *R*_*V*_ at different wavelengths is considered to be induced by the WGM resonance, as confirmed by the PL spectra in Figure S5, which suggests a possible approach to modulating the wavelength-dependent photoresponse of the device.

For comparison, we also tested PTD under identical illumination conditions (Figure S24). It is demonstrated that the *V*_ph_ and *I*_ph_ of PTD also keep linearity with *P*_*λ*_ of incident light, yet the values of *R*_*V*_ and D^*^ are two-order-of-magnitude lower than those of TTD. Figure 4h and Table S2 summarize *R*_*V*_ of our TTD and several other self-driven photodetectors with different working wavelengths based on the PTE effect. The current TTD exhibits a superior broadband response than other PTE detectors made from several materials, including both inorganic and organic materials. It is worth noting that the active material of the current TTD is polycrystalline Te, a much cheaper and conveniently accessible option. Furthermore, the TTD also upholds a prolonged photoresponse consistency even after 180 days in the air atmosphere at room temperature. A marginal decrease in self-driven photocurrent to 92.2% of the initial value (Fig. 4i) attests to its lasting stability and longevity in the air. The durability may be attributed to the presence of Al_2_O_3_ protection layer, which effectively retards the oxidation rate of the active material, i.e., Te layer. The stable SiN_x_ pre-strained layer in the TTD also leads to excellent structural stability for future applications.

### Polarized imaging ability of TTD

**Fig. 5:**
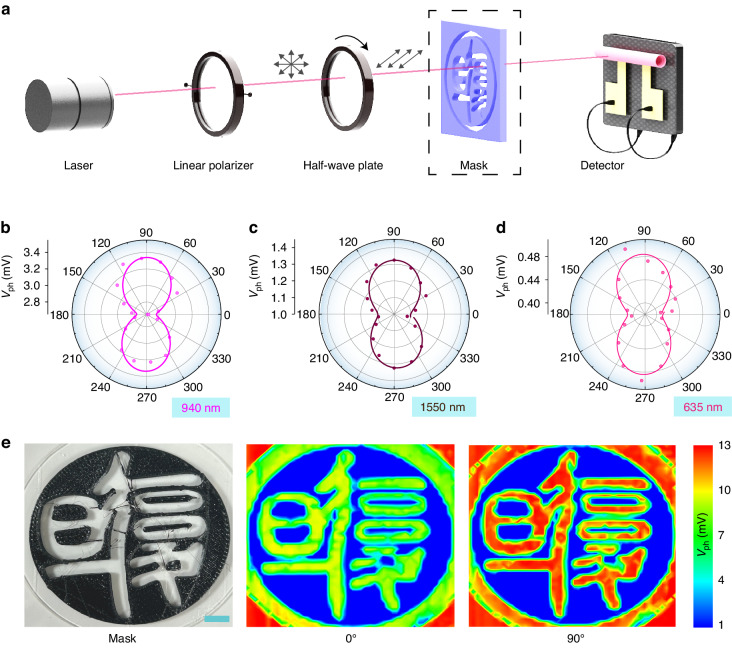
Polarization detection and imaging ability of TTD. **a** Schematic diagram of the polarization detection system. **b**–**d** Polar plots of measured *V*_ph_ of the TTD under polarized light of 940, 1550, and 635 nm lasers. **e** Photo of the original mask pattern and the detected imaging results attained by the TTD with incident light’s polarization angles of 0° and 90°. Scale bar, 5 mm

Tubular detectors are also considered to be competent platforms for polarization detection. Its specific 3D geometry has been proven to achieve polarization sensitivity thanks to the unique tubular geometry and anisotropy introduced^33^. Herein, Fig. 5a schematically illustrates the polarization detection system, involving a fixed linear polarizer, a tunable half-wave plate, and an optional pattern mask. Incident light becomes linearly polarized light after passing through the linear polarizer, and then a polarization angle α is realized by rotating the half-wave plate with an angle of α/2. The experimental results in Fig. 5b–d suggest that as the polarization angle α of the incident light increases under different wavelengths, the value of *V*_ph_ oscillates periodically. The polarization-resolved *V*_ph_ can be well fitted by a sine function (Figure S25). The calculated dichroic ratios of TTD for 940, 1550, and 635 nm lasers are 1.20, 1.21, and 1.19, respectively. It is worth noting that no such polarized response can be observed in PTD (Figure S26), indicating that the polarization detection of TTD is dominated by the tubular geometry. Given the polarization-sensitive characteristics of highly sensitive TTD, we applied the patterned mask to the polarization detection system to explore its potential for polarization imaging. The images in Fig. 5e were detected by single-pixel sensing in the air atmosphere, and they clearly recover the original pattern, exemplifying an outstanding imaging ability of TTD. Meanwhile, a striking variation is obtained under incident light with polarization angles of 0° and 90°. The excellent performance of TTD, including high compatibility, enhanced photoresponse, and remarkable polarization sensitivity, indicates its potential for advanced on-chip broadband photodetection. In addition, TTD with polarized light detection capability can be expected to support 3D imaging by establishing a relationship between polarization characteristics of reflected light and 3D contour.

## Discussion

In summary, we elucidated the multiple field interaction in the self-rolled structure containing a thermoelectric Te layer and developed a powerful technical route to build up a high-performance PTE detector. The TTD, in the form of a tubular structure, was fabricated by self-rolling of nanomembrane which is compatible with existing semiconductor technology. The self-rolled nanomembrane and the corresponding sandwich “SiN_x_-Te-SiN_x_” structure effectively enhances the optical absorption and thermal localization, leading to a significantly enhanced PTE effect which was verified both theoretically and experimentally. Under structural modulation, we meticulously explored the positive linear correlation between rolling rotations and *R*_*V*_ resulting from the light trap effect in the multi-layered nanomembrane. For broadband detection, TTD achieves the highest *R*_*V*_ of 252.13 V W^−1^, D^*^ of 1.48 × 10^11^ *Jones* under 940 nm laser irradiation. Remarkably, self-driven *V*_ph_ in the TTD is approximately 307 times greater than in the PTD. Moreover, TTD manifests outstanding omnidirectional detection, polarization-dependent detection, and imaging characteristics originating from its tubular geometry. This self-rolled structure effectively localizes the optical and heat energies for high-performance photodetection, and we believe the integration of desired functional materials with this 3D on-chip detector should fulfill more advantageous application potentials in the future.

## Materials and Methods

### Fabrication of TTDs

A 20 nm Ge layer was deposited onto the thermal silicon oxide wafer (500 nm SiO_2_ layer on Si wafer) by E-beam evaporation (Lab 18) as the sacrificial layer. SiN_x_ was then deposited by inductively coupled plasma chemical vapor deposition (ICP-CVD, PlasmaPro 100) at 13.56 MHz as the pre-strained layer. Specifically, 20 nm compressive SiN_x_ was deposited at the pressure of 12 mTorr (RF power 0 W, ICP power 20 W, SiH_4_:N_2_ = 13.5:10) and 20 nm tensile SiN_x_ was deposited at the same pressure of 12 mTorr (RF power 10 W, ICP power 20 W, SiH_4_: N_2_ = 13.5: 10). Applying 99.9% Te target (from ZhongNuo Advanced Material Technology Co., Ltd), 30 nm Te layer was deposited by magnetron sputtering (Kurt J Lesker PVD 75) at room temperature (60 sccm Ar, DC power10 W). Then, samples were treated by rapid thermal process system (AccuThermo AW410) under different temperatures for 10 min. 1 μm thick photoresist layer (AZ5214) was spin-coated, and platforms were patterned for isolation (Microwriter ML3) and reactive ion etching (RIE, Trion T2) was then used for 2 min at a pressure of 35 mTorr (30 sccm CF_4_ and 1 sccm O_2_ flow) and RF power of 60 W. 15 nm palladium (Pd) and 65 nm gold (Au) layers were deposited by E-beam evaporation and patterned by photolithography as electrodes. ~ 5 nm Al_2_O_3_ layer was deposited by ALD at 120 °C for protection. Etching windows were opened by a photolithography process. XeF_2_ vapor was applied to selectively etch the sacrificial Ge layer by Memsstar Xenon DiFluoride. The entire structure is schematically shown in Figure S27.

### Optical and thermal simulation

All simulation results including electrical field distribution and temperature distribution were obtained by COMSOL Multiphysics. To reduce the extra computing burdens of the different smallest units, TTD structure was simplified as a tubular structure (inner radii was set to be 16 μm) with 5 periods of Te/SiN_x_ bilayers (thickness of each layer was set to be 50 nm). The boundary condition was programmed as the perfect matching layer. For optical simulation, the refractive indexes of Te and SiN_x_ were set to be 4.92 and 1.98, respectively^46,47^. For thermal simulation, thermal conductivities of Te and SiN_x_ were respectively set to be 2.16 and 2.35 W m^−1^ K^−1^, and heat capacities at constant pressure were respectively set to be 613 and 800 J kg^−1^ K^−1^. Corresponding densities were set to be 3000 and 6250 kg m^−3^, respectively^17,48,49^.

### Materials analysis and structural characterizations

Seebeck coefficient of Te film was measured by Seebeck coefficient analysis system (Cryoall CTA-3). Hall mobility was obtained on Hall effect testing instrument (Sadhudesign SM6800). XRD analysis was performed by Bruker D8 Advance. XPS curves were recorded by Thermo Kalpha. Raman characterizations were carried out on Renishaw inVia Qontor Raman spectrometer with a 100× objective lens. Infrared absorption spectrum was tested by FTIR (Bruker VERTEX 70). UPS spectra were conducted on ESCALAB 250Xi by MeLab (www.micetech.cn). The structures of TTDs were observed by SEM (Zeiss Sigma 300) and optical microscopy (Motic-BA310). After a Pt layer was deposited for surface protection, FIB was utilized to fabricate a sample lamella from TTD. HHADF images were collected by STEM (JEOL ARM200F) at 200 kV.

### Photoresponse mapping and optoelectronic characterization of TTD

The measurement of I-V curves with light illumination at different positions was carried out under irradiation of 940 nm laser with the power density of 1500 μW cm^−2^. The line scan was applied under the illumination of the 940 nm laser with a power density of 900 μW cm^−2^. The photocurrent mapping was applied under the illumination of the 940 nm laser. All photoresponse measurement results including photocurrent mapping, photovoltage line scanning, and other optoelectronic characterizations were collected by MStarter 200 probe station, Keysight B2902B, and lock-in amplifier (OE1022, SSI-USA LLC). During broadband detection, the illumination sources used were 450, 520, 635, 940, and 1550 nm lasers (MW-BL), 4.6 μm laser (MStarter), and 10.6 μm laser (CO_2_ laser, LZ-100).

### Supplementary information

Supplementary Information

## Data Availability

The data that support the findings of this study are available from the corresponding author upon request.
