# Supplementary material for: Enhanced photothermoelectric conversion in self-rolled tellurium photodetector with geometry-induced energy localization
Source: Light Sci Appl. 2024 Jul 4;13:153. doi: 10.1038/s41377-024-01496-0 (PMC11224300; doi:10.1038/s41377-024-01496-0)
Supplement: Supplementary file 1 — Supplementary Information [file 41377_2024_1496_MOESM1_ESM.pdf]

Supplementary Information for

Enhanced photothermoelectric conversion in self-rolled tellurium photodetector with geometry-induced energy localization

Jiayuan Huang[1,2,3], Chunyu You[1,2,3], Binmin Wu[1,2,3], Yunqi Wang[1,2,3], Ziyu Zhang[1,2,3], Xinyu Zhang[1], Chang Liu[1,2,3], Ningge Huang[1,2,3], Zhi Zheng[1,2,3], Tingqi Wu[4], Suwit Kiravittaya[5], Yongfeng Mei[1,2,3,6], and Gaoshan Huang[1,2,3*]

[1]Department of Materials Science & State Key Laboratory of Molecular Engineering of Polymers, Fudan University, Shanghai 200438, China

[2]Yiwu Research Institute of Fudan University, Yiwu 322000, Zhejiang, China

[3]International Institute of Intelligent Nanorobots and Nanosystems, Fudan University, Shanghai 200438, China

[4]ShanghaiTech Quantum Device Lab, ShanghaiTech University, Shanghai 200120, China

[5]Department of Electrical Engineering, Faculty of Engineering, Chulalongkorn University, Bangkok, Thailand

[6]Shanghai Frontiers Science Research Base of Intelligent Optoelectronics and Perception, Institute of Optoelectronics, Fudan University, Shanghai 200438, China

* Corresponding author. Email: gshuang@fudan.edu.cn.

Table S1. Testing results of Seebeck coefficient of Te layer at room temperature.

**Temperature (°C)**	**Resistivity (μΩ m)**	**Resistance (mΩ)**	**Seebeck coefficient (μV K^{-1})**
27.67	386390.17	1457094235.33	2593.11
28.30	376220.23	1418742945.00	2672.72
28.60	366163.29	1380817784.33	2632.55

Table S2. Comparison of photovoltage responsivities of PTE detectors with different working wavelengths reported in recent literature.

Working wavelength (nm)	Responsivity (V W^{-1})	Material	Ref.
405	1.69	$EuBiSe_3$	[1]
450	4.1	Ag_2Te/Te/Ag	[2]
450	24.17	Te (TTD)	This work
520	39.52	Te (TTD)	This work
532	0.13	SnSe	[3]
532	0.85	$SrTiO_3$	[4]
550	0.25	Bi/Au	[5]
635	64.24	Te (TTD)	This work
785	0.15	Single-wall carbon tube/PEDOT: PSS	[6]
808	0.48	4% Co: BiCuSeO	[7]
830	4.4	$LaAlO_3$/$SrTiO_3$	[8]
940	252.13	Te (TTD)	This work
1064	5.5	$HfTe_5$	[9]
1550	12.2	Graphene	[10]
1550	6	Graphene	[11]
1550	3.5	Graphene	[12]
1550	81.62	Te (TTD)	This work

Note S1. Bandwidth-response curve analysis.

The relationship between the photogenerated voltage and frequency of the device can be expressed by the following formula:[13]

$$V(f) = \frac{V_0}{\sqrt{1 + 4\pi^2 f^2 \tau^2}}$$

Here, V_0 represents the value of photovoltage measured under continuous irradiation, f represents the corresponding test frequency, and τ represents the response time. In Figure S9, the gray line is the fitting curve according to this formula. Here, the frequency value f_C corresponding to 0.707 V_0, namely the -3 dB bandwidth, is 205 Hz. And τ is calculated to be 760 μs, which is consistent with the experimental result in Fig. 1f.

Note S2. Absorption spectrum analysis.

The Tauc plot is calculated on the basis of the FTIR absorption spectrum to determine the optical bandgap:[14]

$$(\alpha h v)^{1/r} \propto (h v - E_{\mathrm{g}})$$

Here, α is the absorption coefficient, h is the Planck constant, v is the frequency, E_{g} is the optical bandgap energy, r is 2 in the case of indirect interband transition.

Note S3. UPS spectrum analysis.

The secondary electron cutoff spectrum in the inset of Fig. 2f displays that the Fermi level (E_F) is -2.28 eV, followed by:[15]

$$E_{\mathrm{F}} = hv - E_{\mathrm{cutoff}}.$$

Here, E_{cutoff} refers to the binding energy. Based on the valence-band spectrum in Fig. 2f, the valence band maximum (VBM) position of Te is calculated to be -2.48 eV. Considering the optical band gap of 0.37 eV, the conduction band minimum (CBM) is estimated to be -2.11 eV.

Note S4. Strain in TTDs calculated by Raman peak shift.

As the lattice expands, E_2 and A_1 modes obviously shift, as shown in Fig. 2g. The strain therein thus can be estimated by the shift of Raman mode ($\Delta\omega$), which is related to the shift of phonon frequency:[16]

$$\Delta\omega = \frac{1}{2\omega_0}(p + 2q) \times \varepsilon$$

where ε is the strain in Te and ω_0 refers to ω_{A_1} and ω_{E_2} of unstrained Te. The p and q are linear interpolations that can be extrapolated from previous research.[17] Here, $(p + 2q)_{\omega_{A_1}}$ is $2.44 \times 10^5\ s^{-2}$ and $(p + 2q)_{\omega_{E_2}}$ is $1.70 \times 10^5\ s^{-2}$. The compressive strain in the TTDs is calculated to be 0.35% or 0.50% by using the shift of A_1 or E_2 mode under 532 nm laser illumination.

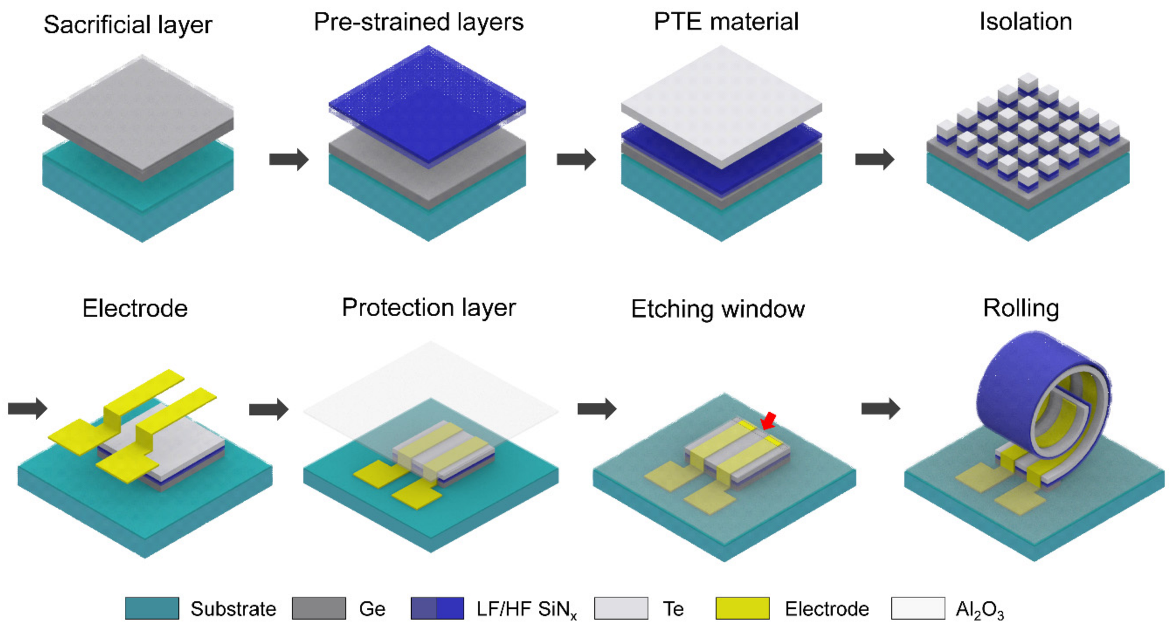

Figure S1. Flow chart of the fabrication process of TTD: (1) Sacrificial Ge layer is deposited by E-beam evaporation. (2) Pre-strained SiN_x layer is deposited by ICP-CVD. (3) PTE material (Te) layer is deposited by magnetron sputtering. (4) Isolation is conducted by photolithography and reactive ion etching. (5) Pd/Au electrode is deposited by E-beam evaporation. (6) Protection Al_2O_3 layer is deposited by ALD. (7) Etching window is created by photolithography and etching. (8) Sacrificial Ge layer is removed by XeF_2 etching and multilayered nanomembrane is rolled up.

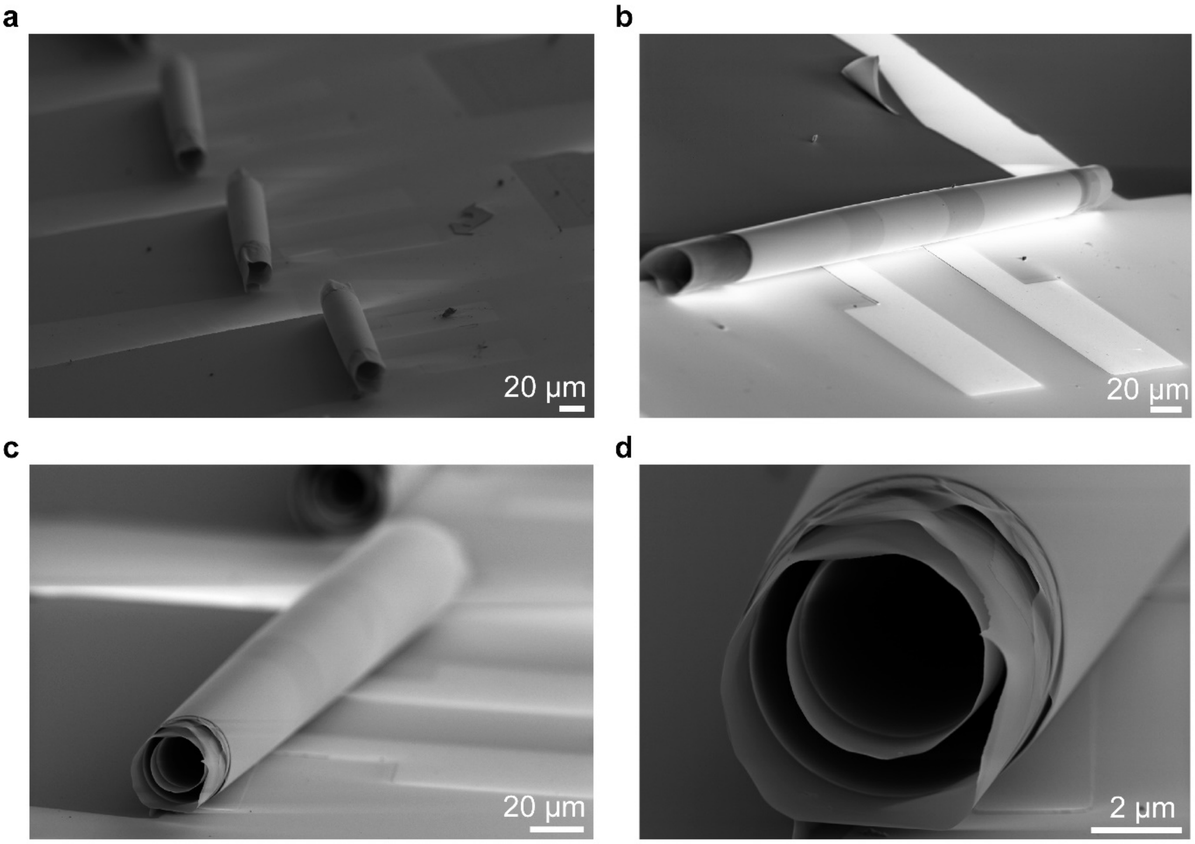

Figure S2. a) SEM image of TTDs array. b-d) SEM images of TTDs with different diameters.

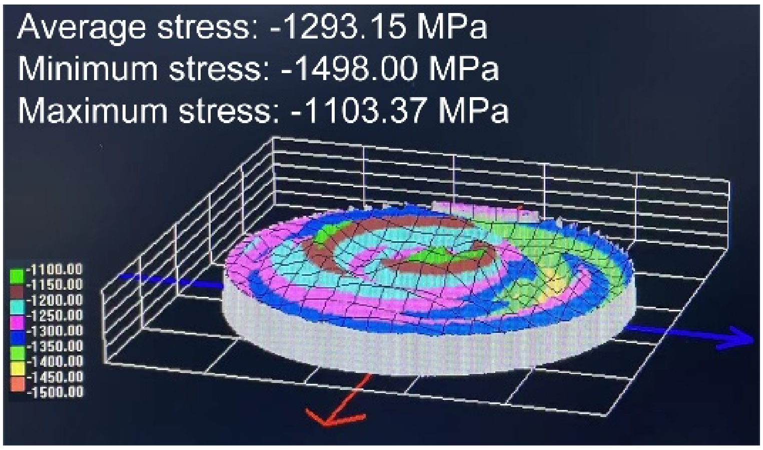

Figure S3. Stress difference in dual-layer SiN_x nanomembrane on a 4-inch wafer. Average stress difference reaches -1293.15 MPa.

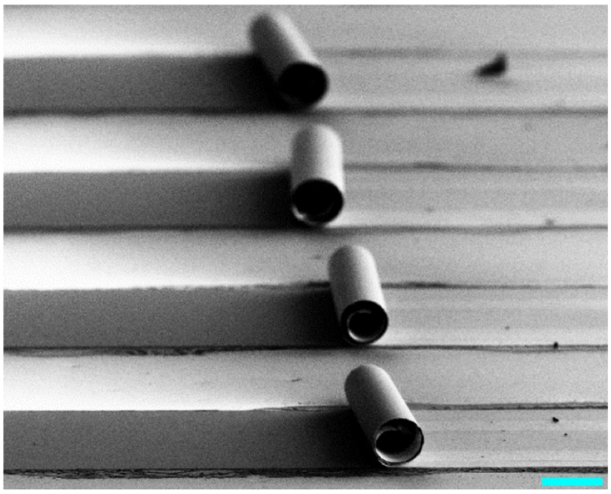

Figure S4. SEM image of self-rolled TTD devices array. Scale bar: 50 μm.

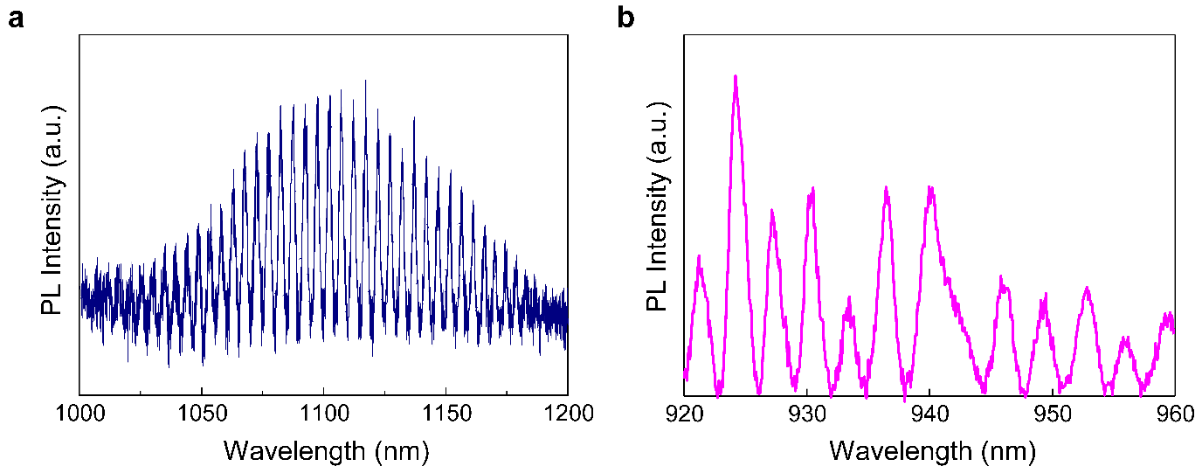

Figure S5. PL spectra of TTD in the ranges of: a) 1000-1200 nm and b) 920-960 nm. The PL spectra were collected under the illumination of 785 nm laser.

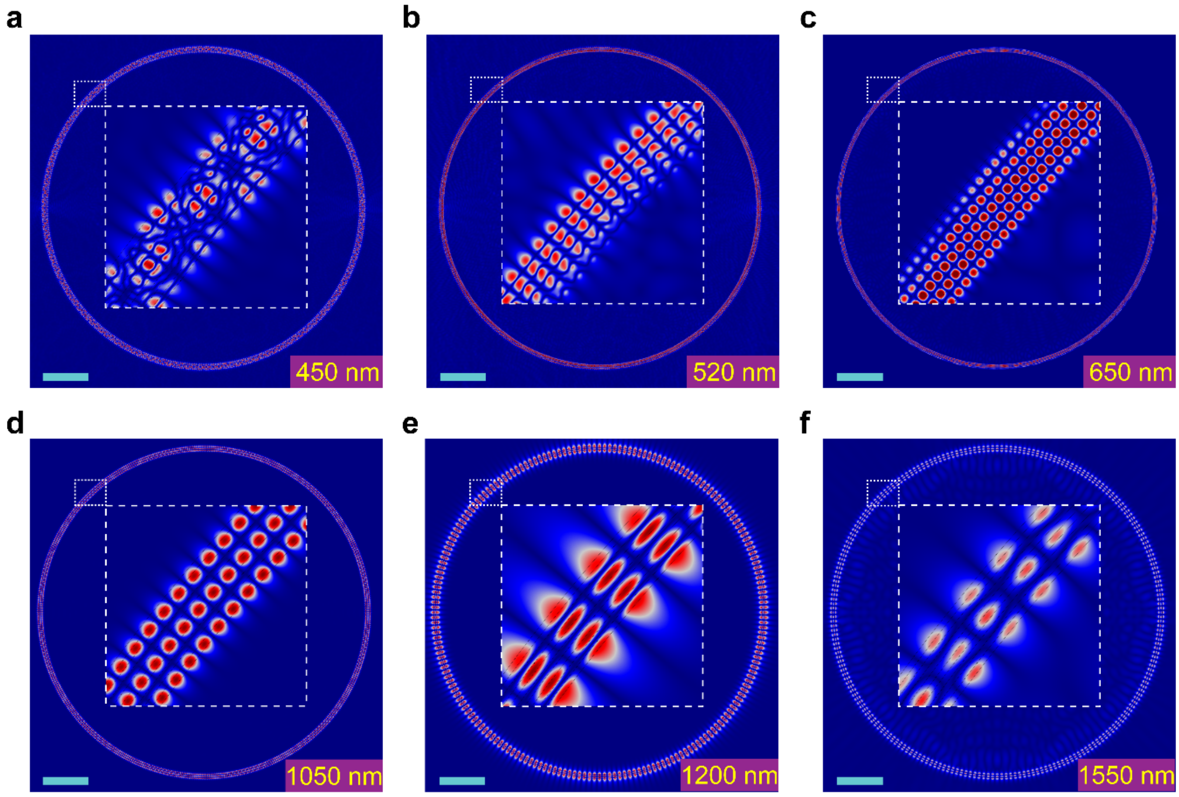

Figure S6. Simulated field distribution at wavelengths of a) 450, b) 520, c) 650, d) 1050, e) 1200, and f) 1550 nm. Scale bars are 5 μm. The insets show the details of the field distribution.

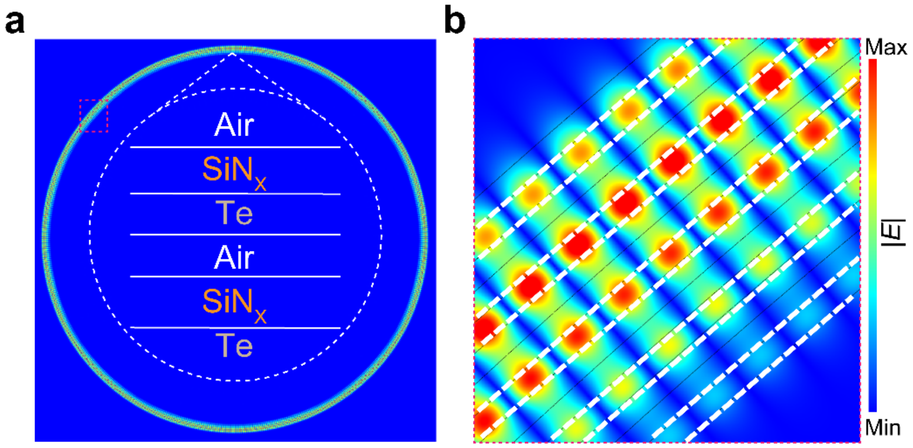

Figure S7. a) Simulated electrical field distribution (940 nm) in the tube wall with air gaps added. b) Enlarged view of the electrical field distribution in the multi-layered tube wall. The Te layers are marked with white dashed lines.

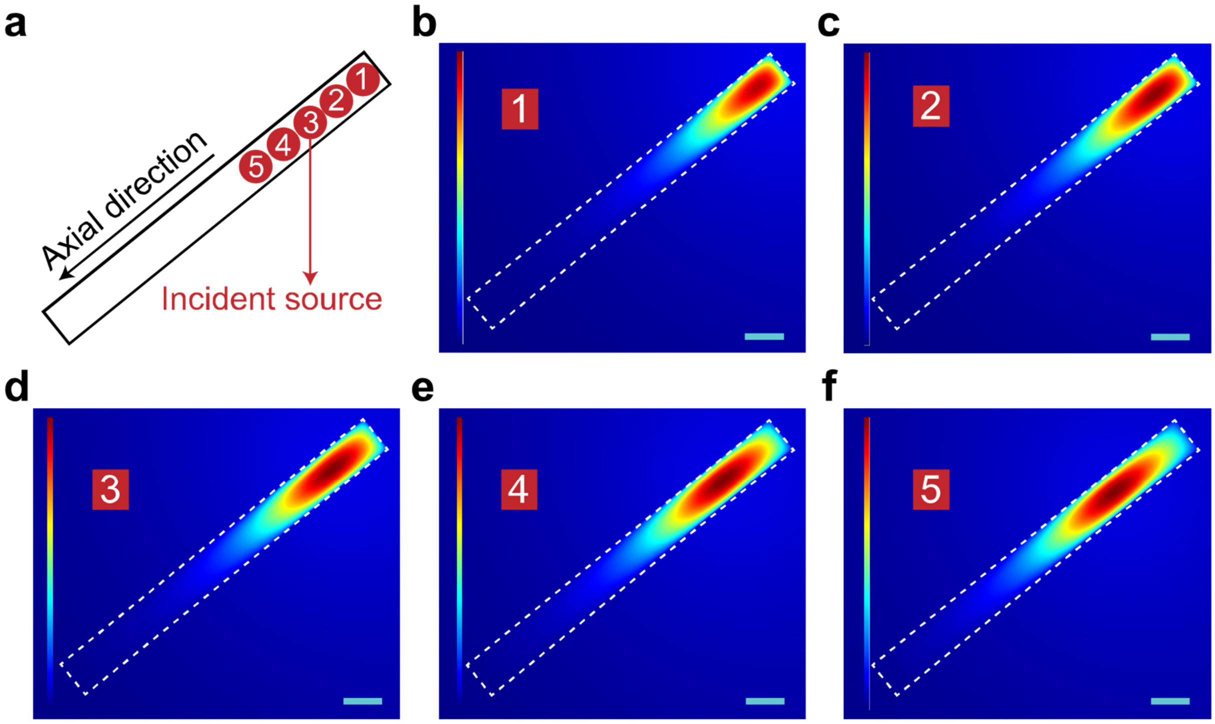

Figure S8. a) Schematic diagram of the light illumination at five different spots (positions 1-5) along tube axis. b-f) Simulated temperature distribution with light illumination at different spots corresponding to a). Scale bars are 30 μm.

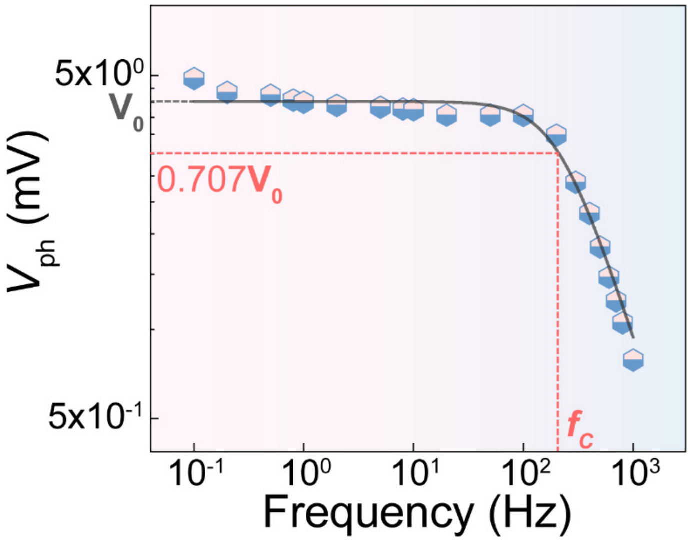

Figure S9. Bandwidth-response curve of a TTD irradiated by a 940 nm laser.

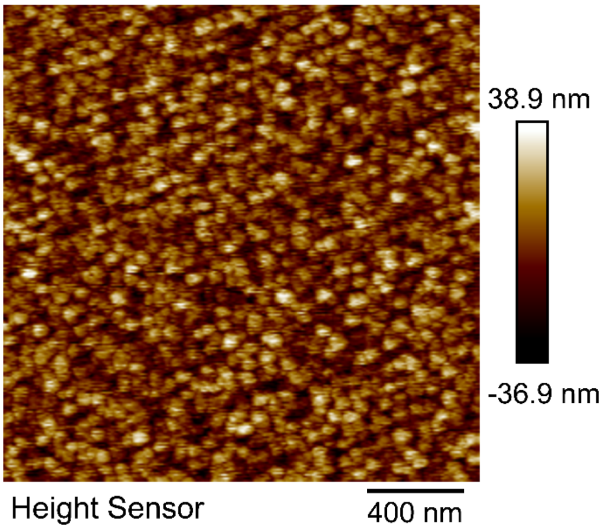

Figure S10. AFM image of Te layer prepared by magnetron sputtering.

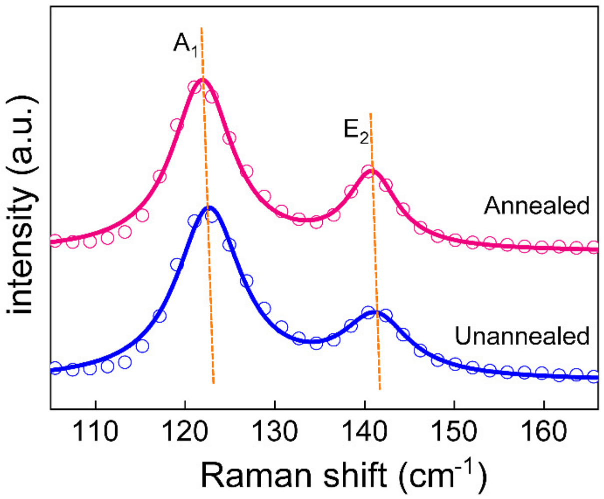

Figure S11. Raman spectra of Te layer before and after being annealed at 150 °C. A 532 nm laser was used as the light source.

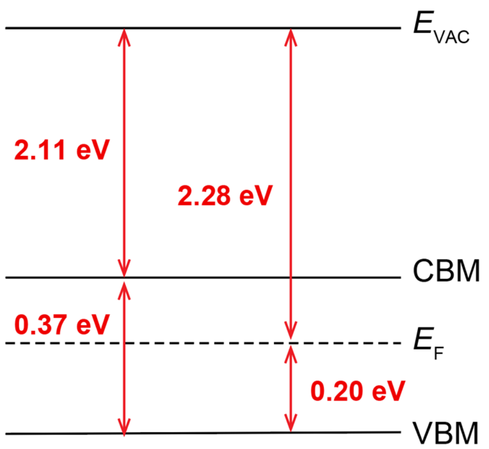

Figure S12. Schematic diagram of the band structure of Te layer.

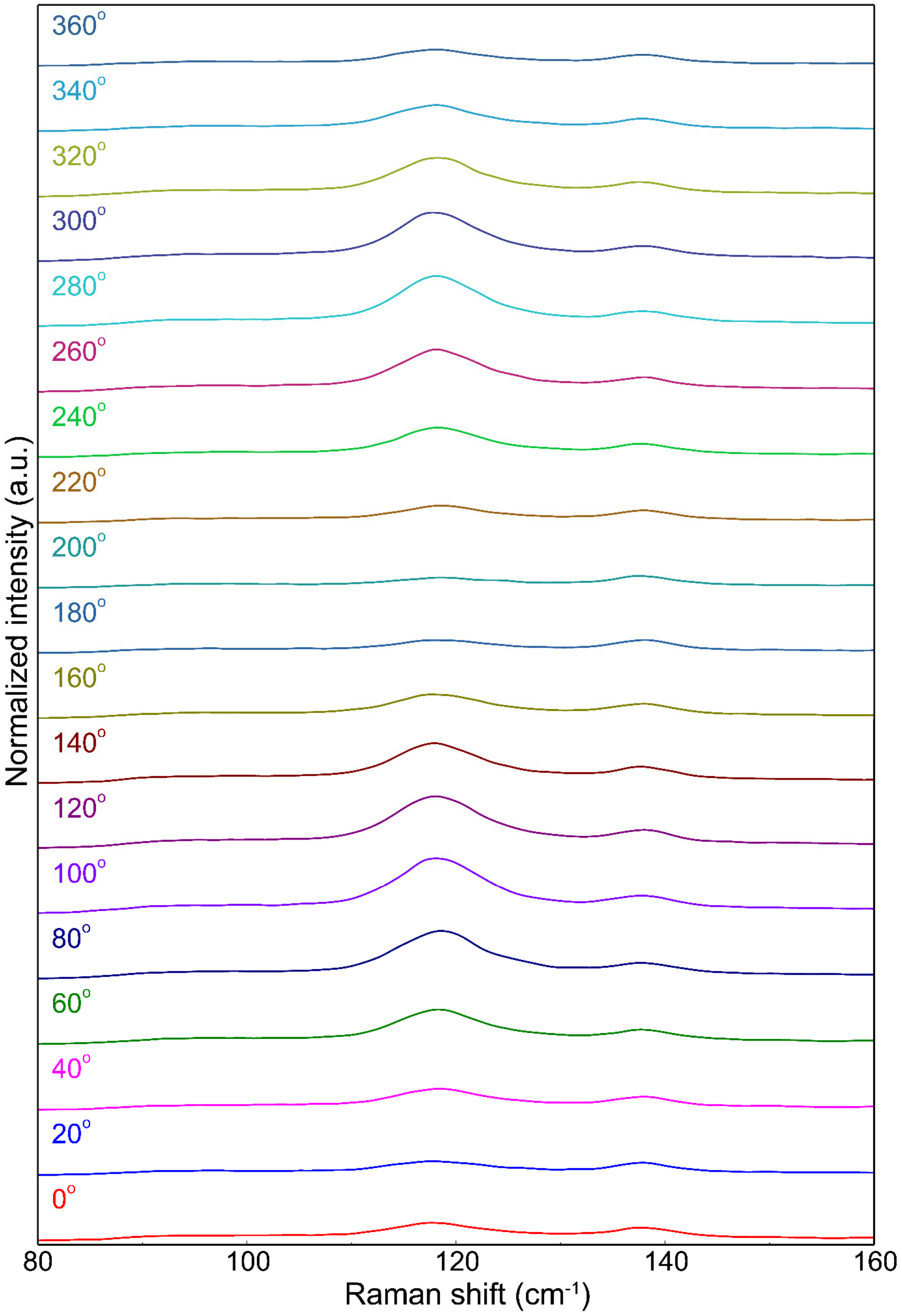

Figure S13. Raman spectra of flat Te layer with polarized angle ranging from 0° to 360°. A 532 nm laser was used as the light source.

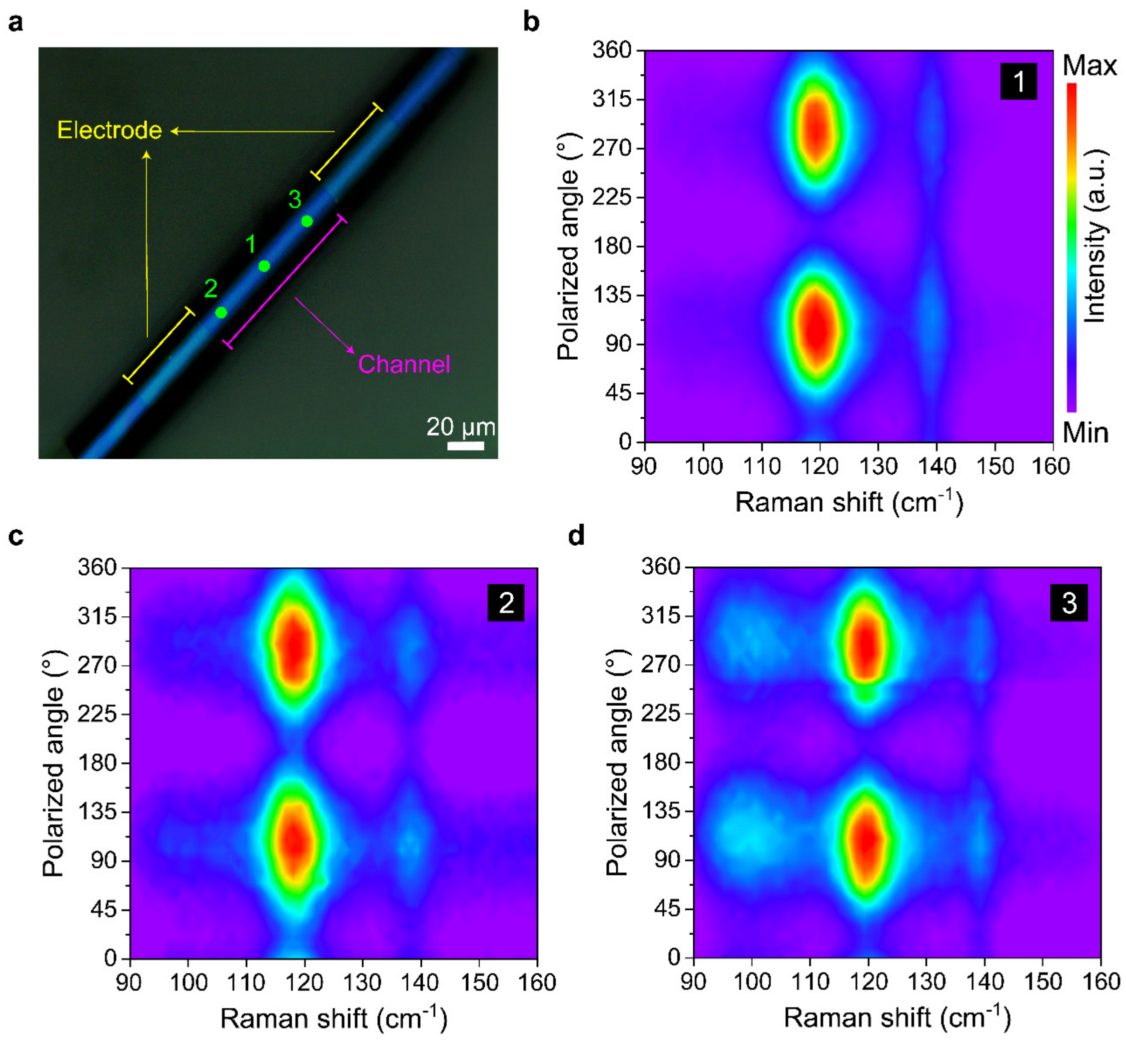

Figure S14. a) Optical microscopy image of a TTD and spots 1-3 are labelled. b-d) Measured polarized Raman spectra with light illumination at spots 1-3. A 532 nm laser was used as the light source.

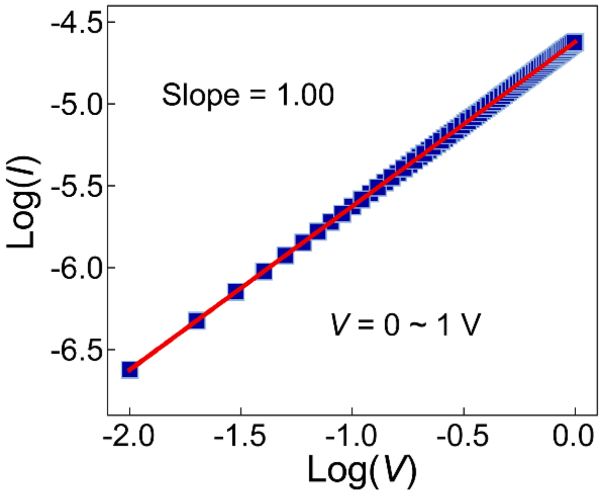

Figure S15. Log(*I*)-Log(*V*) curve demonstrating a linear relationship, ranging from 0 to 1 V.

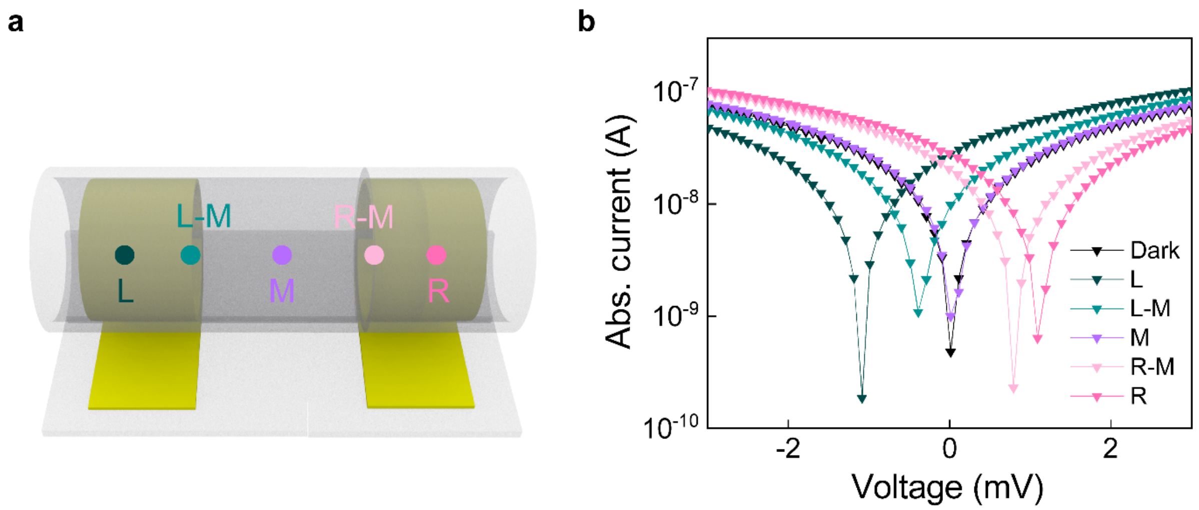

Figure S16. a) Schematic diagram of photoresponse measurement for TTD with different illuminated positions: left (L), left-medium (L-M), medium (M), right-medium (R-M), and right (R). b) Corresponding *I-V* curves. The light source is a 940 nm laser with the power density of 1500 μW cm^{-2}.

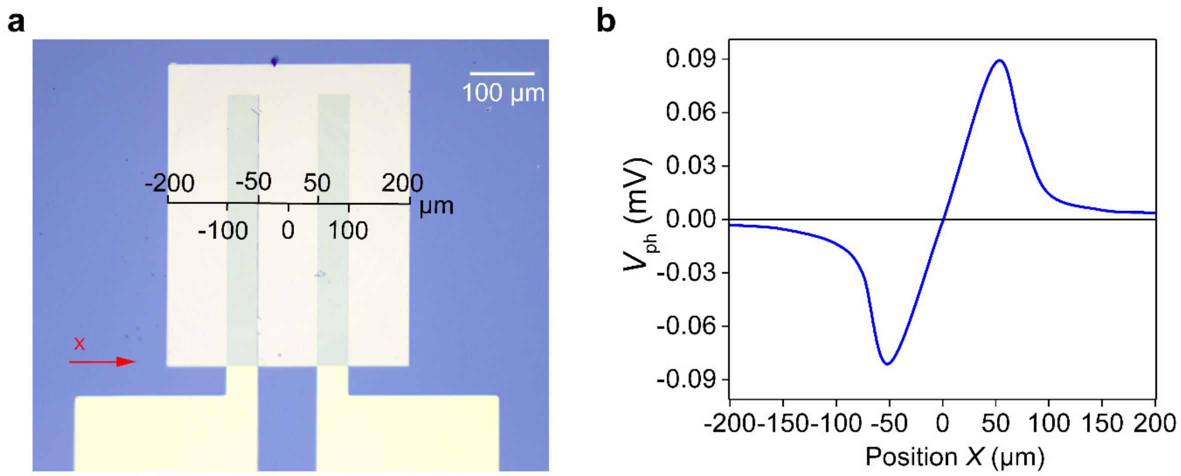

Figure S17. a) Optical microscopy image of the PTD. b) Measured photovoltage of PTD along the horizontal direction. The light source is a 940 nm laser with the power density of 1500 µW cm^{-2}.

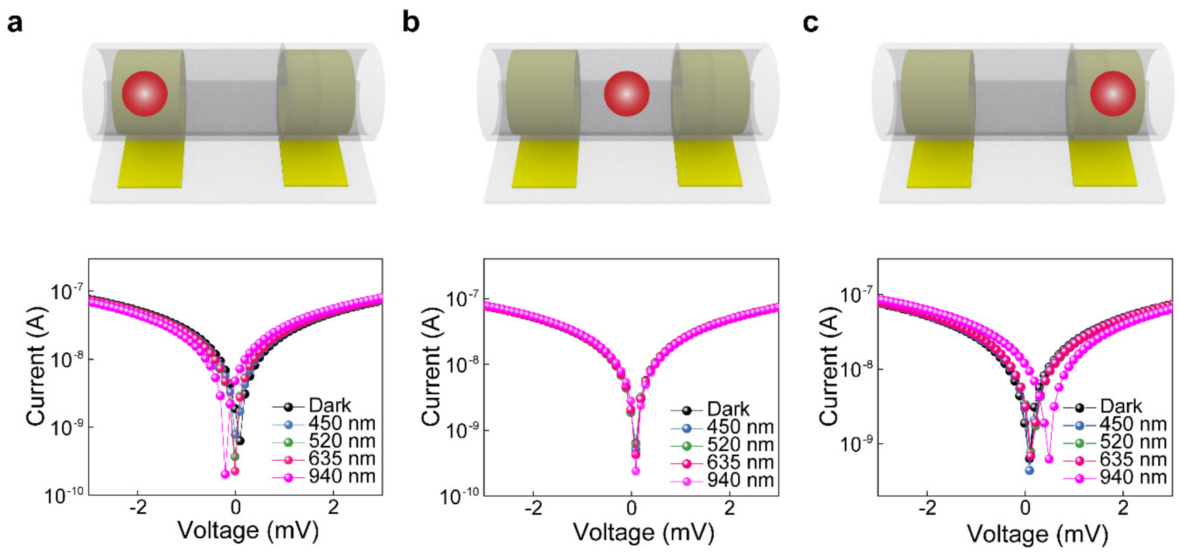

Figure S18. *I*-*V* curves of TTD illuminated with different wavelengths at corresponding positions: a) Position $X > 0$, b) Position $X = 0$, and c) Position $X < 0$. The light source is a 940 nm laser with the power density of 900 μW cm^{-2}.

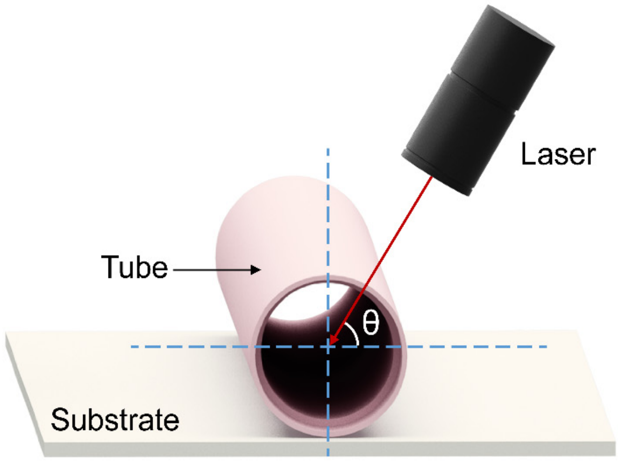

Figure S19. Schematic diagram of the angle-resolved characterization system.

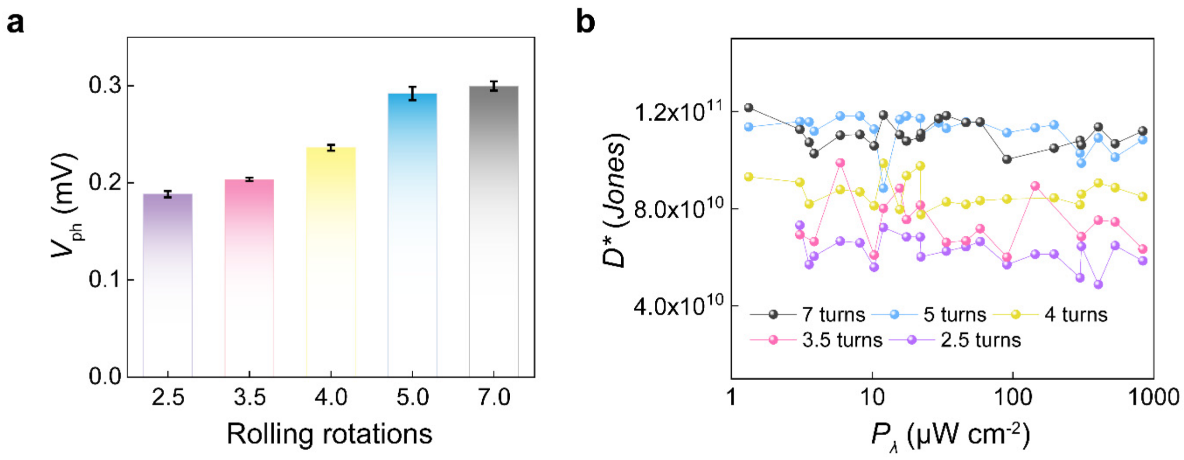

Figure S20. a) Photovoltage of TTDs with different rolling rotations under 940 nm laser illumination. b) Detectivity of TTDs with different rolling rotations under 940 nm laser illumination.

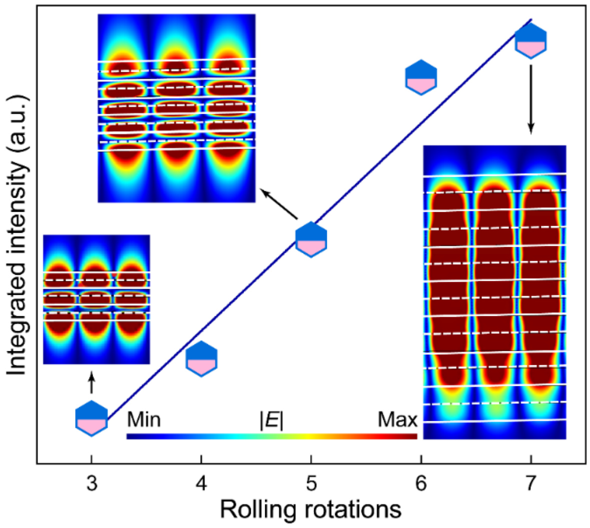

Figure S21. Integrated electric field intensity as a function of rolling rotations with the same incident light source of 940 nm. Inset: the enlarged panels of simulated field distributions of self-rolled structures with 3, 5 and 7 rotations.

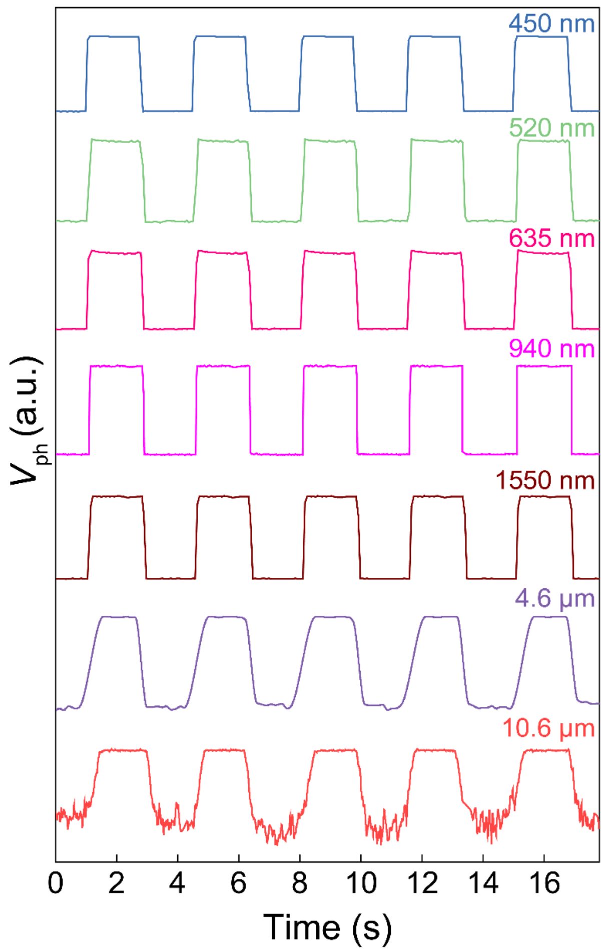

Figure S22. V_{ph}-t curves of the TTD with pulsed illuminations of 450 nm, 520 nm, 635 nm, 940 nm, 1550 nm, 4.6 μm, and 10.6 μm lasers at zero bias.

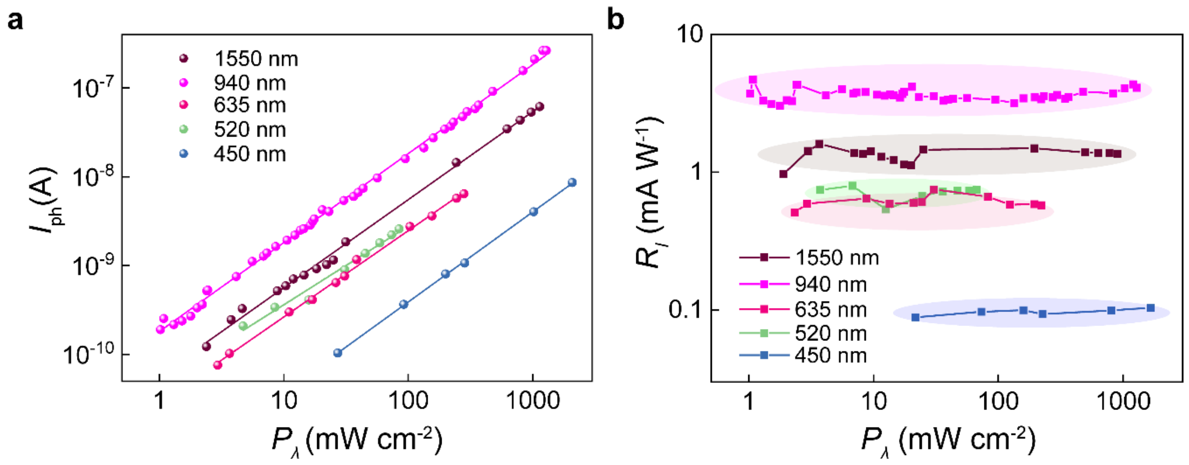

Figure S23. a) Dependence of I_{ph} on P_λ of lasers with different wavelengths. b) The relationships between R_I and P_λ with different illumination wavelengths.

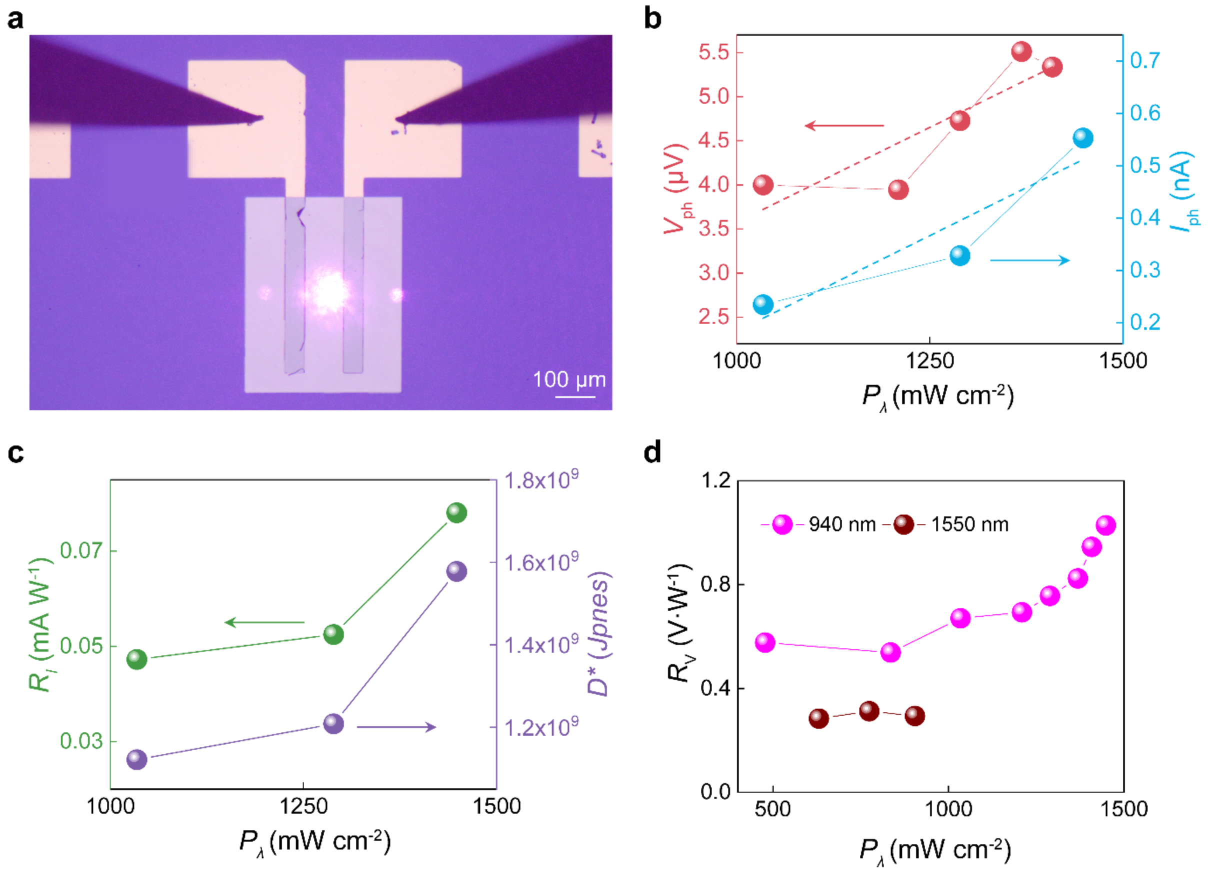

Figure S24. a) Optical microscopy image of a PTD. b) V_{ph} and I_{ph} as functions of P_λ under 940 nm laser illumination. c) R_I and D^* as functions of P_λ under 940 nm laser illumination. d) Calculated R_V under different illumination wavelengths.

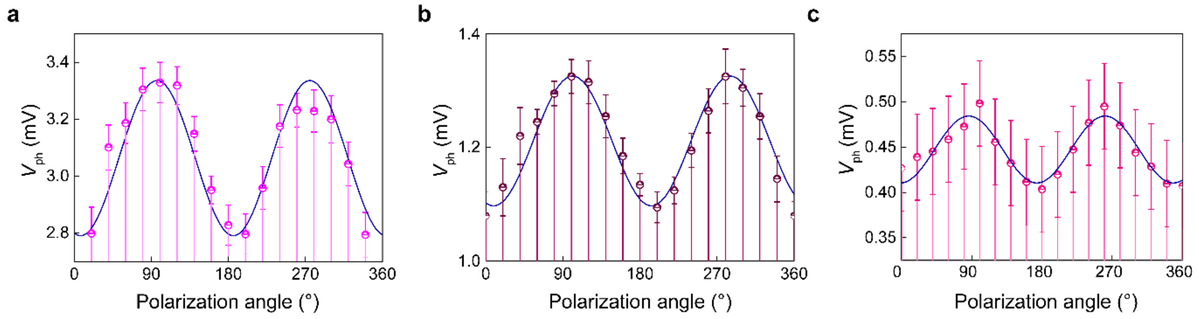

Figure S25. Polarization-resolved photovoltages under incident lights with different wavelengths of (a) 940, (b) 1550, and (c) 635 nm. The laser power densities are set to be 900 μW cm^{-2}.

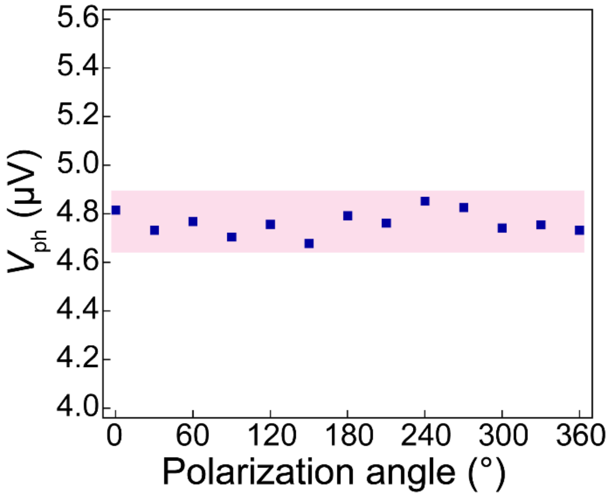

Figure S26. Measured V_{ph} of PTD illuminated by a 940 nm laser with different polarization angles. The power density is set to be 900 mW cm^{-2}.

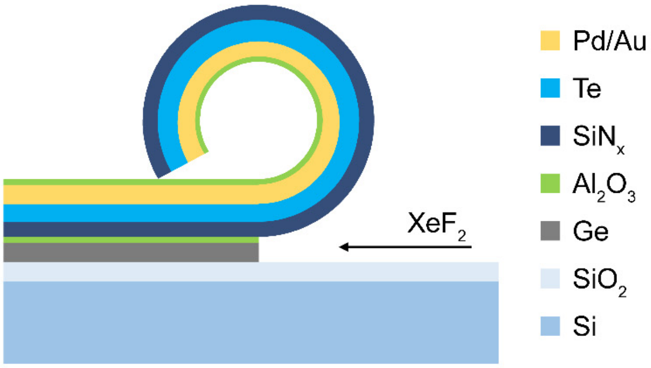

Figure S27. Schematic diagram of multilayered structure of TTD. Thermally oxidized silicon wafer includes Si and SiO_2. Ge is used as sacrificial layer, Al_2O_3 as protection layer, SiN_x as prestrained layer, Te as PTE active material, and Pd/Au as electrode.

References

[1] Wang, Y., et al. Ultrabroadband, sensitive, and fast photodetection with needle-like $EuBiSe_3$ single crystal. *ACS Photonics* **6**, 895-903 (2019).

[2] Wang, R., et al. Manipulating nanowire structures for an enhanced broad-band flexible photothermoelectric photodetector. *Nano Letter* **22**, 5929-5935 (2022).

[3] Zhong, Y., et al. High-quality textured SnSe thin films for self-powered, rapid-response photothermoelectric application. *Nano Energy* **72**, 104742 (2020).

[4] Lu, X., Jiang, P. & Bao, X. Phonon-enhanced photothermoelectric effect in $SrTiO_3$ ultra-broadband photodetector. *Nature Communication* **10**, 138 (2019).

[5] Monshat, H., Liu, L. & Lu, M. A narrowband photo-thermoelectric detector using photonic crystal. *Advanced Optical Materials* **7**, 1801248 (2018).

[6] Wang, J., Xie, Z., Liu, J. A. & Yeow, J. T. W. Design of room-temperature infrared photothermoelectric detectors based on CNT/PEDOT:PSS composites. *Journal of Materials Chemistry C* **10**, 15105-15113 (2022).

[7] Wang, F., et al. Enhanced photothermoelectric detection in Co:BiCuSeO crystals with tunable Seebeck effect. *Optics Express* **30**, 8356-8365 (2022).

[8] Dai, W., et al. Large and reconfigurable infrared photothermoelectric effect at oxide interfaces. *Nano Letter* **19**, 7149-7154 (2019).

[9] Niu, Y., et al. Ultrabroadband, fast, and flexible photodetector based on $HfTe_5$ crystal. *Advanced Optical Materials* **8**, 2000833 (2020).

[10] Muench, J. E., et al. Waveguide-integrated, plasmonic enhanced graphene photodetectors. *Nano Letter* **19**, 7632-7644 (2019).

[11] Miseikis, V., et al. Ultrafast, zero-bias, graphene photodetectors with polymeric gate dielectric on passive photonic waveguides. *ACS Nano* **14**, 11190-11204 (2020).

[12] Marconi, S., et al. Photo thermal effect graphene detector featuring 105 Gbit s^{-1} NRZ and 120 Gbit s^{-1} PAM4 direct detection. *Nature Communication* **12**, 806 (2021).

[13] Fang, H. & Hu, W. Photogating in low dimensional photodetectors. *Advanced Science* **4**, 1700323 (2017).

[14] Gao, S., et al. Catalyst-free synthesis of sub-5 nm silicon nanowire arrays with massive lattice contraction and wide bandgap. *Nature Communication* **13**, 3467, (2022).

[15] Zou, T., et al. High-performance solution-processed 2D P-type WSe_2 transistors and circuits through molecular doping. *Advanced Materials* **35**, 2208934, (2023).

[16] Songmuang, R., Jin-Phillipp, N., Mendach, S.& Schmidt, O. G. Single rolled-up SiGe/Si microtubes: Structure and thermal stability. *Applied Physics Letter* **88**, 021913 (2006).

[17] Du, Y., et al. One-dimensional van der Waals material tellurium: Raman spectroscopy under strain and magneto-transport. *Nano Letter* **17**, 3965-3973 (2017).
